# Serotype I and II Feline Coronavirus Replication and Gene Expression Patterns of Feline Cells—Building a Better Understanding of Serotype I FIPV Biology

**DOI:** 10.3390/v14071356

**Published:** 2022-06-22

**Authors:** Sarah Cook, Diego Castillo, Sonyia Williams, Christine Haake, Brian Murphy

**Affiliations:** 1 Graduate Group Integrative Pathobiology, School of Veterinary Medicine, University of California, Davis, CA 95616, USA; 2 Department of Pathology, Microbiology, and Immunology, School of Veterinary Medicine, University of California, Davis, CA 95616, USA; ldcastillo@ucdavis.edu (D.C.); sywilliams@ucdavis.edu (S.W.); bmurphy@ucdavis.edu (B.M.); 3 School of Veterinary Medicine, University of California, Davis, CA 95616, USA; cjhaake@ucdavis.edu

**Keywords:** feline infectious peritonitis, FIP, serotype, cell receptor, coronavirus, viral replication

## Abstract

Feline infectious peritonitis (FIP) is a disease of domestic cats caused by the genetic variant of the feline coronavirus (FCoV) and feline infectious peritonitis virus (FIPV), currently grouped into two serotypes, I and II. Although serotype I FIPV is more prevalent in cats with FIP, serotype II has been more extensively studied in vitro due to the relative ease in propagating this viral serotype in culture systems. As a result, more is known about serotype II FIPV than the more biologically prevalent serotype I. The primary cell receptor for serotype II has been determined, while it remains unknown for serotype I. The recent development of a culture-adapted feline cell line that more effectively propagates serotype I FIPV, FCWF-4 CU, derived from FCWF-4 cells available through the ATCC, offers the potential for an improved understanding of serotype I FIPV biology. To learn more about FIPV receptor biology, we determined targeted gene expression patterns in feline cells variably permissive to replication of serotype I or II FIPV. We utilized normal feline tissues to determine the immunohistochemical expression patterns of two known coronavirus receptors, ACE2 and DC-SIGN. Lastly, we compared the global transcriptomes of the two closely related FCWF-4 cell lines and identified viral transcripts with potential importance for the differential replication kinetics of serotype I FIPV.

## 1. Introduction

Feline infectious peritonitis (FIP) is a generally fatal coronaviral disease of domestic cats caused by a genetic variant of the feline coronavirus (FCoV), referred to as the FIP virus (FIPV) [1,2]. FIP is a systemic immune-mediated, inflammatory disease variably characterized by fever, ascites accumulation, fibrinous exudate, pyogranulomatous perivasculitis, and high mortality [3,4,5]. Virus-associated inflammation may be confined to the abdominal cavity but can also involve the thoracic cavity, lymph nodes, brain, or eye [4].

FCoV is subclassified into two biotypes, feline enteric coronavirus (FECV) and feline infectious peritonitis virus (FIPV), and further classified into two serotypes (serotype I and serotype II), currently taxonomically grouped as a single species of *Alphacoronavirus* based on genomic sequencing [6]. The two distinct serotypes are classified based on variation in the humoral response stemming from marked sequence differences between spike proteins [7]. The coronavirus spike (S) protein serves as the docking ligand for the cell receptor, thereby defining the cellular tropism and tissue-based distribution of the virus. Cellular tropism has been investigated for FIPV through in vitro recombination studies and the generation of viral chimeras to map regions of the S protein that may be responsible for these differences in cell tropism [8,9]. Serotype II FIPV is the evolutionary result of a recombination event between feline coronavirus and canine enteric coronavirus, resulting in a chimeric FCoV encoding the canine coronavirus *spike* gene [10,11]. However, this taxonomic organization has been challenged by the suggestion that serotype I and II FCoV be redefined as distinct viruses based on distinguishing differences in spike protein structure and function and the pivotal roles that spike plays in biological and clinical outcomes [12].

In nature, serotype I FCoVs, including FIPV I, are more prevalent than FIPV II viruses and are associated with the majority (80–95%) of naturally occurring cases of FIP in cats (serotype I and II FIPVs are hereafter referred to as FIPV I or FIPV II) [13,14,15]. However, the in vitro propagation of FIPV I isolates in tissue culture systems has proven to be problematic, impeding forward progress in tissue-culture-based virology and pathogenesis investigations. While the primary cellular receptor utilized for cell entry by FIPV II has been identified as feline aminopeptidase N (feAPN/CD13), the primary cellular receptor utilized by FIPV I remains to be determined [16,17,18,19]. There are conflicting results regarding the possibility of feAPN serving as a primary cell receptor for FIPV I. Tresnan et al. (1996) found that non-permissive mouse or hamster cells, transduced to stably express feAPN, became permissive to both FIPV I and II replication [16]. In contrast, Hohdatsu et al. (1998) found that when applying feAPN antibodies to FIPV permissive cells (FCWF-4), the cells were no longer infectable by FIPV II, while FIPV I was still able to infect the antibody-treated cells [19]. Similarly, Dye et al. (2007) found out through the use of retroviral pseudotypes bearing the type I or type II S glycoprotein that type I S glycoprotein failed to transduce cell lines known to express feAPN (Crandall-Reese feline kidney cells (CRFK), *Felis catus* whole fetus 4 (FCWF-4), and feline kidney fibroblast cells) [18]. These experimental inconsistencies, along with inconsistencies in reported abilities to propagate FIPV I in a number of cell lines (including CRFK and FCWF-4), call into question whether feAPN can be absolutely eliminated as a putative FIPV I receptor.

FIPV II readily replicates in several different feline cell lines (e.g., CRFK and FCWF-4) [20]. The FIPV I Black strain (also referred to as TN406 and hereafter referred to as Black I) was originally isolated from an experimental case of FIP and is one of the few tissue-culture-adapted FIPV I strains [21]. Black I has been successfully propagated in a spontaneously immortalized cell line derived from fetal feline airway epithelial cells (AK-D) [22]. However, FIPV targets feline monocytes/macrophages in vivo and not airway epithelium. Thus, a more biologically relevant cell line may provide better insight into the naturally occurring disease in cats [23,24,25]. The cell line FCWF-4 is a feline macrophage-like cell that is permissive for FIPV I replication [20]. FCWF-4 cells are described as “macrophage-like” based on studies demonstrating nonspecific esterase expression (a cytochemical marker of macrophages), the phagocytic properties of the cells, and the detection of Fc receptors on the FCWF-4 surface membrane [26]. Although permissive to FIPV I, viral replication has been shown to be reduced (10^4^ vs. 10^6^ TCID50/mL) and delayed (96 vs. 24 h post-infection) in FCWF-4 cells relative to FIPV II replication [20,22,26,27,28,29]. Recently, an FCWF-4-derived cell line established at Cornell University (herein referred to as FCWF-4 CU) demonstrated dramatically improved Black I replication kinetics, with rapidly increased viral titers relative to FCWF-4 obtained directly from the American Type Culture Collection (hereafter referred to as FCWF-4 ATCC) [22]. Possible reasons for improved replication in FCWF-4 CU cells relative to FCWF-4 ATCC cells include variations in antiviral interferon responses, differences in expression and density of the presently undetermined FIPV I receptor [22], or differences in the expression of host cell proteases necessary for coronavirus cell entry or activation.

Different alphacoronaviruses and betacoronaviruses share a discrete collection of receptors for cell entry (Table 1) [16,30,31,32,33]. Known cellular receptors of coronaviruses include APN, dendritic cell-specific intercellular adhesion molecule-3-grabbing nonintegrin (DC-SIGN), angiotensin-converting enzyme 2 (ACE2), and dipeptidyl-peptidase 4 (DPP4/CD26). The C-type lectin, DC-SIGN, has been shown to enhance cell entry by FIPV II acting as a co-receptor [24,34]. One study demonstrated that the induction of DC-SIGN expression in CRFK cells renders them permissive to Black I infection, suggesting that serotype I strains might be dependent upon lectins as a co-receptor and that CRFK cells might express the primary receptor at low levels [24].

Here, we characterize FIPV I and II replication in three feline cell lines (CRFK, FCWF-4 ATCC, FCWF-4 CU) and in primary feline monocyte-derived macrophages (feMDMs). Based upon the known coronaviral receptor redundancy, we explored the targeted gene expression patterns in feline cell lines and feMDMs and hypothesized that these expression patterns would facilitate the identification of the FIPV I receptor. Using antibodies targeting two of the investigated feline-specific receptor proteins (DC-SIGN and ACE2), we utilized immunohistochemistry to characterize the feline tissue and cellular distribution patterns of these putative coronaviral receptors. Finally, we hypothesized that the global transcriptome for the two closely related FCWF cell lines would inform the identification of putative viral receptors or other cellular features important for the differential replication kinetics of FIPV I.

## 2. Materials and Methods

### 2.1. FIPV Inoculum for In Vitro Experiments

FIPV II (WSU-79-1146; GenBank DQ010921) was propagated and viral infectivity was quantified in CRFK cells using a biological plaquing assay (median tissue culture infectious dose, TCID50); viral RNA was quantified using reverse transcription real-time polymerase chain reaction (RT-qPCR) as previously described [35]. FIPV I (Black I) and FCWF-4 CU cells were kindly donated by Dr. Susan Baker (Loyola University Chicago), originally obtained from Cornell University College of Veterinary Medicine (Dr. Edward Dubovi). Briefly, Black I virus was propagated in FCWF-4 CU cells by inoculating a confluent 75 cm^2^ tissue culture flask (Corning, Corning, NY, USA) with Black I virus at a multiplicity of infection (MOI) of 0.1 in 3 mL of minimal essential media (MEM; Sigma, St. Louis, MO, USA) lacking fetal bovine serum (FBS) and incubated for 1 h at 37 °C, tilting the flask every 15 min. The inoculum was then removed, and 10 mL of MEM + 2% FBS was added to the flask and incubated at 37 °C for an additional 24 h before collecting the cultured supernatant and centrifuging the sample at 775× *g* rpm for 5 min. The resulting supernatant was aliquoted into 100 or 500 µL volumes and frozen at −80 °C. The determination of the FIPV serotype was confirmed using serotyping primers (FCoV I S For, FCoV II S For, FCoV I and II S Rev; nFCoV I S For, nFCoV II S For, and nFCoV I and II S Rev) targeting a portion of the *spike* gene previously published and listed in Table 2 [36]. All additional primers utilized in this research project can be found in Table 2.

### 2.2. RNA Processing

RNA for all experiments was isolated using Invitrogen’s PureLink RNA Mini Kit and subsequently treated to remove possible DNA contamination using TURBO DNAse from the same manufacturer. Samples in which cDNA was to be generated were reverse transcribed using Applied Biosystem’s High-Capacity RNA-to-cDNA Kit, including reactions lacking the reverse transcriptase (RT) enzyme (RT-control) (Thermo Scientific).

### 2.3. Black I and WSU-79-1146 Titers across Cell Types

To determine the relative replication efficiencies of Black I and WSU 79-1146 (FIPV II) in the various cell types (CRFK, FCWF-4 ATCC, FCWF-4 CU, and feline monocyte-derived macrophages (feMDMs)), TCID50 was determined by a biological plaquing assay as previously described and published [35]. Goat synovial membrane (GSM) cells were utilized as a non-permissive negative control cell line. GSM cells were originally derived from fetal goat tissues and were cultured in DMEM/10% FBS [37]. Due to challenges in obtaining complete monolayers of feMDMs necessary for the colorimetric plaquing assay, the replication of Black I and WSU 79-1146 viruses in feMDM cells was determined using RT-qPCR. Using density/centrifugation-based separation, feMDMs were derived from feline peripheral whole blood by isolating peripheral blood mononuclear cells (PBMCs) from approximately 5 to 7 mL of feline whole blood obtained from purposefully bred specific pathogen-free cats from the University California Feline Nutrition Center (UC Davis) in accordance with UC Davis Nutrition Center IACUC protocol and guidelines. Freshly isolated feline PBMCs were resuspended and plated in RPMI-1640 (HyClone) media supplemented with 10% fetal bovine serum (FBS; Gemini, New York, NY, USA), 10% feline serum (Equitech Bio, Kerrville, TX, USA), 100 U/mL penicillin and 0.1 mg/mL streptomycin (Gibco, Waltham, MA, USA), 2 mM GlutaMAX (Gibco), 20 ng/mL recombinant human granulocyte macrophage colony-stimulating factor (GM-CSF; R&D Systems, Minneapolis, MN, USA), and 10 ng/mL recombinant human interleukin-4 (IL-4; Gibco). Culture media was exchanged for fresh media every two days for eight to ten days to allow for adherence and macrophage differentiation based on cytomorphology (“fried-egg” morphology) and the immunofluorescent labeling of the macrophage marker CD14 (BioRad, TUK4; method is described below, Hercules, CA, USA) [38]. Feline MDMs were cultured in a 24-well tissue culture plate and subsequently infected with either Black I or WSU 79-1146 at MOI 0.1 in triplicate wells. Infected cells were incubated for approximately 36 h, at which time cell-associated total RNA was isolated and processed as previously described (Section 2.2).

The copy number for all target genes was determined using Applied Biosystem’s QuantStudio 3 Real-Time PCR System and PowerUp SYBR Green Master Mix, following the manufacturer’s protocol for a 10 µL reaction. Cycling conditions were as follows: 50 °C for 2 min and 95 °C for 2 min, followed by 40 cycles of 95 °C for 15 s, respective annealing temperature for 30 s (see Table 2), and 72 °C for 1 min. The final step included a dissociation curve to evaluate the specificity of primer binding. All reactions were performed in triplicate with a water template as a negative control and plasmid DNA as a positive control. Copies of target gene cDNA were normalized to 10^6^ copies of feline *GAPDH* cDNA based on standard curves generated in our laboratory. Results were graphed using Prism 9 (GraphPad), and a Student’s *t*-test was performed to determine the significance of differences in the mean viral titer.

### 2.4. Quantification of FIPV and Cellular Gene Expression by RT-qPCR

Four feline cell types (feMDM, FCWF-4 ATCC, FCWF-4 CU, and CRFK) were evaluated for gene expression patterns of known coronavirus cellular receptors. FeMDMs were cultured as described above. CRFK cells were grown in DMEM/10% FBS. FCWF-4 ATCC and CU cells were grown in MEM supplemented with 1% pen/strep, 1% L-glutamine, 1% HEPES (1M), 1% sodium pyruvate solution, 1% MEM non-essential amino acid solution, and 10% FBS, followed by sterile filtration through a 0.22-micron membrane filter.

In order to assess gene expression patterns of known alpha- and betacoronavirus receptors (ACE2, DC-SIGN, fAPN, and DPP4), the above-listed feline cell types were propagated in tissue culture plates, and total cell-associated RNA was isolated and processed as previously described (Section 2.2). Except for the listed and previously published serotyping primers, all qPCR cycling conditions for all target genes were the same, aside from annealing temperatures, which can be found in Table 2. The copy number for all target genes was determined using Applied Biosystem’s QuantStudio 3 Real-Time PCR System and PowerUp SYBR Green Master Mix, following the manufacturer’s protocol for a 10 µL reaction. All reactions were performed in triplicate with a water template as a negative control and plasmid DNA as a positive control. Copies of target gene cDNA were normalized to 10^6^ copies of feline *GAPDH* cDNA based on standard curves generated in our laboratory.

### 2.5. CD14 Immunofluorescence

CD14 immunofluorescence was performed to provide supportive evidence of a histiocytic (macrophage) phenotype for feMDM cells. FeMDM cells were cultured in a 24-well tissue culture plate, as described above. Feline MDM cells were gently washed three times with phosphate-buffered saline (PBS) for 5 min per wash. Cells were then fixed with 100% methanol for 10 min and then incubated with a blocking solution containing 5% bovine serum albumin (tris-buffered saline with 0.1% Tween 20 and 5% bovine serum albumin), rocking it for 1 h at room temperature. Cells were washed three times with PBS and incubated with a primary antibody, mouse anti-human CD14, at a dilution of 1:500 for one hour at room temperature. Cells were washed three times with PBS before incubation with the conjugated secondary antibody (FITC anti-mouse IgG; Vector Laboratories) at 1:1000 dilution for 30 min at room temperature. After washing with PBS three more times, one drop of SlowFade Gold Antifade reagent (Invitrogen, Waltham, MA, USA) containing DAPI was applied to the well. The cells were then assessed visually using fluorescence microscopy with GFP and DAPI light cubes (Life Technologies EVOS digital inverted microscope and cell imaging system). This procedure was repeated for FCWF-4 ATCC, FCWF-4 CU, and CRFK cells.

### 2.6. Determining the Systemic Distribution of ACE2 and DC-SIGN in Feline Tissues

In order to characterize the feline tissue distribution patterns of ACE2 and DC-SIGN proteins, immunohistochemical staining was performed on formalin-fixed paraffin-embedded (FFPE) tissues derived from a healthy cat. The feline FFPE tissues had been previously archived in our laboratory from an unrelated study.

Heat-induced epitope retrieval (HIER) was performed using citrate buffer (Dako, Carpinteria, CA, USA) before incubation with the primary rabbit polyclonal anti-ACE2 antibody (Abcam) and human monoclonal anti-DC-SIGN antibody (NIH AIDS Reagent Program; 9′ E9A8). Immunoreactivity was visualized using the NovaRED Substrate Kit that utilizes horseradish peroxidase (Vector). Slides were assessed by visual inspection performed by a board-certified veterinary pathologist (S. Cook). The antibodies utilized were chosen due to previous use in feline cells or formalin-fixed tissue, and appropriate expression was confirmed using positive control tissues (feline kidney for ACE2 and lymph node for DC-SIGN (dendritic cells)) [34,39] The ACE2 antibody had been previously published for use in cats and ferrets [40]. The DC-SIGN antibody had been previously published for use in vitro in feline cell lines (CRFK) [34]. A complete set of feline tissues was reviewed, and immunohistochemical staining was recorded for tissue type, cell type expressing the protein, and subcellular localization of immunoreactivity and graded on a semi-quantitative scale of 0 to 3 for intensity of staining. A grade of 0 indicates a complete lack of staining; 3 indicates intense dark staining. Subcellular localization of immunoreactivity was characterized as membranous, cytoplasmic, or both; there was no nuclear immunoreactivity identified. Membranous refers to immunoreactivity primarily localized to the cell membrane while cytoplasmic refers to signals limited to the cytoplasm of the cell. In some cases, immunoreactivity was both membranous and cytoplasmic.

### 2.7. Gene Expression Profiles of FCWF-4 ATCC and FCWF-4 CU Cell Lines Using RNAseq

Three individual cell cultures representing different passage numbers of both FCWF-4 ATCC and FCWF-4 CU cells were individually processed for total cell-associated RNA using the PureLink RNA mini kit (Invitrogen) and eluted into 50 µL of molecular grade water (Invitrogen). Quality and quantity of the RNA were determined using NanoDrop (ThermoScientific, Waltham, MA, USA), and approximately 10 µg of RNA from each culture was subsequently treated with DNAse (TURBO DNAse; Invitrogen), following the manufacturer’s protocol. The six total cell-associated RNA samples (3 for each cell line) were stored at −80 °C until the time of submission for 3′ Tag-Seq RNA sequencing (RNAseq) at the UC Davis Genome Center as a single batch.

### 2.8. RNAseq

Barcoded 3′Tag-Seq libraries were prepared using the QuantSeq FWD kit (Lexogen, Vienna, Austria) for multiplexed sequencing according to the recommendations of the manufacturer. The fragment size distribution of the libraries was verified via micro-capillary gel electrophoresis on a Bioanalyzer 2100 (Agilent, Santa Clara, CA, USA). The libraries were quantified by fluorometry on a Qubit instrument (LifeTechnologies, Carlsbad, CA, USA) and pooled in equimolar ratios. Ninety-three libraries were sequenced on one lane of a NextSeq500 sequencer (Illumina, San Diego, CA, USA) with single-end 85 bp reads. The sequencing generated more than 4 million reads per library.

HTStream [41] was used to clean raw sequencing reads, removing PhiX sequences, Illumina adapters, poly AT sequences, low-quality regions (average quality less than 20), and reads below 50 base pairs in length. Unique molecular identifiers (UMIs) were also processed using a custom python script and UMI tools to remove PCR duplicates [42]. The remaining reads were then mapped to the *Felis catus* genome (Felis_catus_9.0) using STAR [43], and a counts table was generated using featureCounts [44] and the corresponding Ensembl (version 106) genome annotation. Differential expression analysis was then conducted in R (The R Project for Statistical Computing) [45] with the limma voom pipeline [46,47], which consists of normalization and statistical testing, followed by multiple testing corrections via the Benjamini–Hochberg procedure [48]. Pathway enrichment analysis was also performed, identifying enriched Gene Ontology (GO) terms in topGO [49] (v2.48.0) with the Kolmogorov–Smirnov test [50] and enriched KEGG pathways using the Wilcoxon rank-sum test in KEGGREST (v1.36.0) [51,52]. Heatmaps were prepared from the data matrices of all transcripts and of the differentially expressed genes in R.

## 3. Results

### 3.1. Comparison of Serotype I and II Titers Produced in Each Cell Line

**Figure 1:**
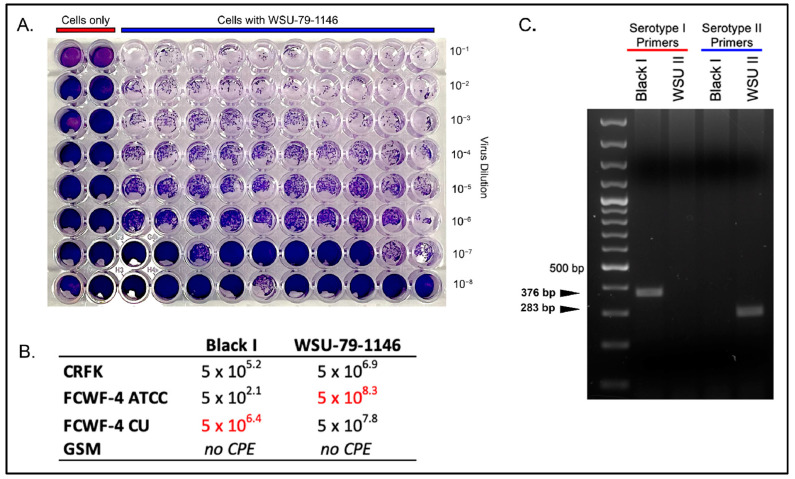
TCID50 results for Black I and WSU-79-1146 in feline cell lines. (**A**) Representative example of a TCID50 plate for WSU-79-1146 grown in CRFK cells. Wells are stained with crystal violet stain to evaluate the presence of cytopathic effect (plaque clearing) across serial dilutions of the virus. (**B**) Summary table of TCID50 values (TCID50/mL) for Black I and WSU-79-1146 grown in four feline cell lines. Numbers highlighted in red represent the maximal TCID50 achieved for each viral serotype. (**C**) Agarose gel electrophoresis image confirming the serotype of the two viral stocks. The nested serotyping primers are listed in Table 1 (FCoV I S For, FCoV II S For, and FCoV I and II S Rev; nested primers are nFCoV I S For, nFCoV II S For, and nFCoV I and II S Rev). The nested primer set was not necessary as PCR amplicons were evident using the initial primer set alone. The expected amplicon size for serotypes I and II is 376 and 283 bp, respectively.

In order to characterize the viral replication of Black I and WSU 79-1146 among the different cell types, biological plaquing assays were performed in 96-well tissue culture plates (Figure 1). Black I replicated to maximal titers in FCWF-4 CU cells (5 × 10^6.4^ TCID50/mL) and less efficiently in CRFK cells (5 × 10^5.2^ TCID50/mL) and demonstrated limited replication in FCWF-4 ATCC cells (5 × 10^2.1^).

Interestingly, WSU 79-1146 replicated most efficiently in FCWF-4 ATCC cells (5 × 10^8.3^ TCID50/mL), although relatively high viral titers were recovered from CRFK cells (5 × 10^6.9^ TCID50/mL) and FCWF-4 CU cells (5 × 10^7.8^ TCID50/mL). These results suggest that CRFK cells may not be the optimal cell line for in vitro propagation of WSU 79-1146 FIPV. Neither virus displayed any evidence of cytopathic effect (plaques) in the non-permissive caprine GSM cell line, consistent with a lack of viral replication (negative control cells).

To genetically confirm the viral serotype, the two viral stocks were “serotyped” using standard PCR targeting spike and the FCoV I S For and Rev primers (listed in Table 2). These PCR reactions yielded the appropriate amplicon sizes of 376 bp (serotype I FIPV) and 283 bp (serotype II FIPV, Figure 1C).

**Figure 2:**
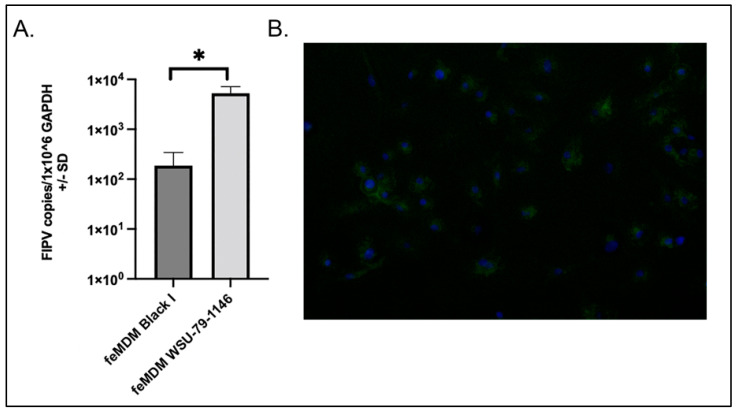
feMDM viral growth patterns and CD14 immunofluorescence. (**A**) Replication of serotype I and II FIPV in feMDM cells was quantified using RT-qPCR and was normalized to the expression of the housekeeping gene, *GAPDH.* Serotype II FIPV (WSU 79-1146) replicated more efficiently than serotype I FIPV (Black I) in feMDM cells (* indicates significance between groups, *p* = 0.0094, Student’s *t*-test). (**B**) Immunofluorescence of CD14 to confirm macrophage differentiation from feline monocytes (green fluorescence). Blue staining represents DAPI staining for cell nuclei. Green fluorescence (FITC) represents membranous CD14 staining.

Due to challenges in producing a tightly confluent monolayer of primary feMDMs in tissue culture wells, a comparison of viral replication between Black I and WSU 79-1146 in feMDMs was obtained by quantifying cell-associated viral RNA normalized to *GAPDH* expression. We found that WSU 79-1146 (FIPV II) replicated more efficiently (showed higher copy numbers) than Black I in feMDMs by a factor of almost two logs (*p* = 0.0094, *t*-test; Figure 2A).

**Figure 3:**
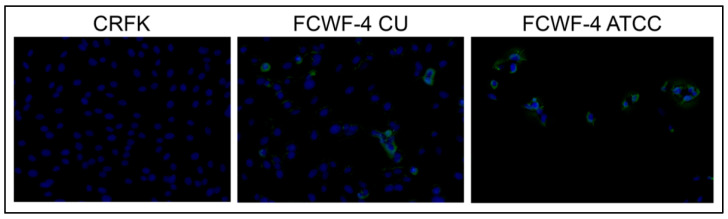
Comparison of cellular CD14 expression via immunofluorescence. CRFK cells demonstrate a lack of CD14 expression, and the two FCWF-4 cell line images have variable membranous expression by FCWF-4 cells (green fluorescence). Blue staining represents DAPI staining for cell nuclei. Green fluorescence represents FITC membranous CD14 staining.

Positive immunofluorescence with macrophage marker CD14 confirmed a macrophage-type phenotype of the infected feMDM cells (green fluorescence, Figure 2B). As FCWF-4 cells have been described as “macrophage-like”, we also investigated the expression of CD14 in these two cell lines (FCWF-4 CU and FCWF-4 ATCC) and in CRFK cells, which are not expected to demonstrate CD14 expression. As expected, membranous CD14 expression was identified in both of the FCWF-4 cell lines but was not identified in CRFK cells (Figure 3).

### 3.2. Cellular Expression of Known Coronavirus Receptors

**Figure 4:**
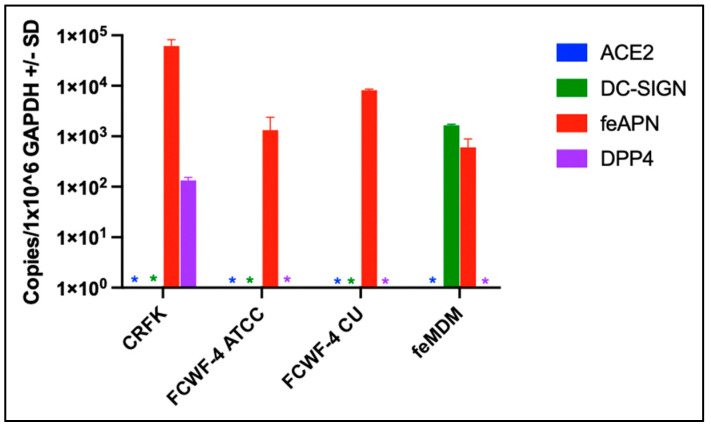
Expression profiles of known coronavirus receptor genes among cell lines permissive to either FIPV I or II tissue-culture-adapted strains, Black I and WSU-79-1146, respectively. Colored asterisks (*) represent undetected gene expression.

Targeted gene expression patterns of known coronavirus receptors in feline cells variably permissive to FIPV replication were determined and normalized to GAPDH expression using RT-qPCR. Feline cells known to efficiently propagate FIPV II include CRFK, FCWF-4 ATCC, FCWF-4 CU, and feline monocyte-derived macrophages [22,53]. We found that gene expression of feAPN was greatest in CRFK cells; however, feAPN expression was pronounced in all four of the examined cell types (Figure 4). Since feAPN serves as the primary cellular receptor for FIPV II, these results are consistent with Figure 1B and Figure 2A (demonstrating efficient replication of FIPV II in all four of these feline cell types).

DC-SIGN expression, the receptor previously demonstrated to serve as an optional co-receptor for FIPV II, was not detected in CRFK cells, consistent with the concept that DC-SIGN is not required for FIPV II cell entry [54]. However, DC-SIGN expression was identified in feMDMs (macrophages serving as host cells for FIPV in vivo). Interestingly, ACE2 expression, which has been suggested as a potential FIPV I receptor [55], was not identified in feMDMs or in either of the FCWF-4 cell lines, yet all three of these cell types are permissive to FIPV I replication (Figure 1 and Figure 2). These results suggest that ACE2 does not serve as the primary cellular receptor for FIPV I. DPP4 expression was identified only in CRFK cells and at low levels. If DPP4 served as a primary FIPV I receptor, its expression would be expected in feMDMs and FCWF-4 CU and FCWF-4 ATCC cells.

### 3.3. Feline Tissues and Cellular Distribution of ACE2 and DC-SIGN Proteins via IHC

The systemic distribution of DC-SIGN and ACE2 proteins in feline tissues was determined using immunohistochemistry. In this study, we found no evidence that ACE2 functions as a primary receptor for either FIPV I or II. However, given the interest in ACE2, the primary receptor of SARS-CoV-2, and the role of DC-SIGN as a co-receptor for FIPV II, we chose to characterize the distribution patterns of these proteins in normal feline tissues.

**Figure 5:**
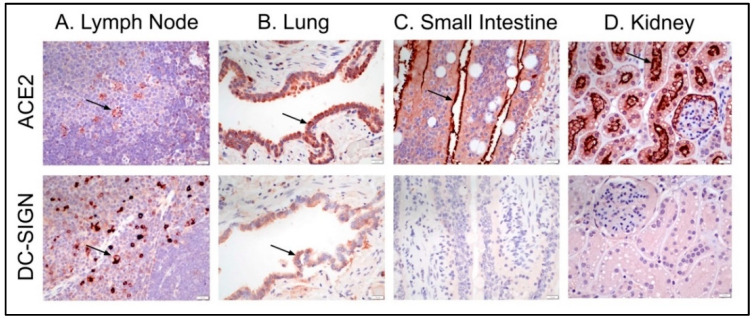
ACE2 and DC-SIGN immunohistochemistry panel of representative tissues. Brown immunostaining represents antigen detection of either ACE2 or DC-SIGN (arrows denote examples of antigen detection). (**A**) Scattered cells with antigen detection for ACE2 in lymphoid follicle germinal centers and paracortex for DC-SIGN. (**B**) Bronchiolar epithelial antigen detection for ACE2 and weaker signal detected for DC-SIGN. (**C**) Small intestinal villous epithelial antigen detection for ACE2, particularly along the apical aspect of epithelial cells and absent antigen detection for DC-SIGN. (**D**) Strong antigen detection for ACE2 within renal cortex tubular epithelium for ACE2 and absent antigen detection for DC-SIGN.

We found that ACE2 was more widely expressed than DC-SIGN in normal feline tissues and cells. ACE2 protein expression was most pronounced in thyroid follicular cells, epithelial cells (particularly tongue, airway, kidney, and small and large intestines), and scattered cells in the intestinal lamina propria. In contrast, DC-SIGN expression (presumably labeling histiocytes based on cell location and morphology) was most pronounced in individualized, scattered cells within the lymph node paracortex, to a lesser extent within the subcapsular sinuses and splenic red pulp, as well as in the intestinal lamina propria (Table 3, Figure 5). The cell labeling with DC-SIGN is presumably labeling histiocytes (dendritic cells and macrophages) based on the location of these cells as well as cell morphology. Dendritic cells are found in tissues involved with immune surveillance and locations in contact with the outside environment, which include lymphoid tissue and the intestinal tract, as we have demonstrated immunohistochemically [56]. Individual cells with ACE2 expression in the lymph node are mostly located within germinal centers, with rare individual cells expressing ACE2 within the paracortex or subcapsular sinuses. ACE2 expression by cells within germinal centers is morphologically consistent with macrophages (Figure 5A). ACE2 expression by macrophages within the spleen and lymph nodes has also been reported in humans [57].

### 3.4. RNAseq Gene Expression Profiles of FCWF-4 ATCC and FCWF-4 CU Cell Lines

RNAseq analyses were performed to determine the global transcriptome of FCWF-4 ATCC and FCWF-4 CU cells. Differentially expressed (DE) genes and the magnitudinal differences in gene expression between these two closely related cell lines were determined. As Black I (FIPV I) replicates with different efficiencies in these two cell lines, we reasoned that identifying DE genes would provide insight into cell factors critical for viral replication (e.g., cell receptors, other cellular factors).

**Figure 6:**
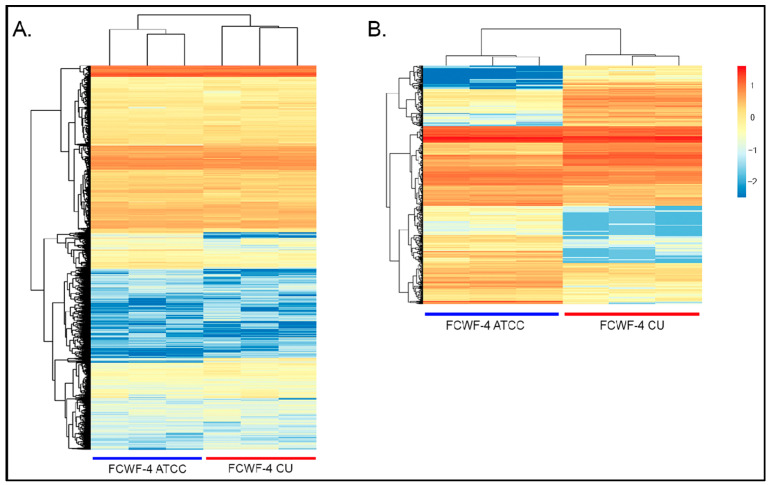
Cluster analysis of differential gene expression. (**A**) Heatmap of 10,036 genes depicting differential gene expression between FCWF-4 ATCC (blue bar) and FCWF-4 CU (red bar) transcriptomes. (**B**) Heatmap of 458 significantly differentially expressed genes between FCWF-4 and FCWF-4 CU cell lines. Each column represents a different cell passage. Both column and row clustering were applied.

In this transcriptome dataset, the expression of 10,036 feline genes was identified, with 458 significantly DE genes (Figure 6). Visual assessment (cluster analysis heatmap) of the overall differential gene expression covering all detected gene transcripts is depicted in Figure 6A. In Figure 6B, the 458 DE genes are depicted. Minor transcriptional variability was identified between cell passage replicates within each cell line.

**Figure 7:**
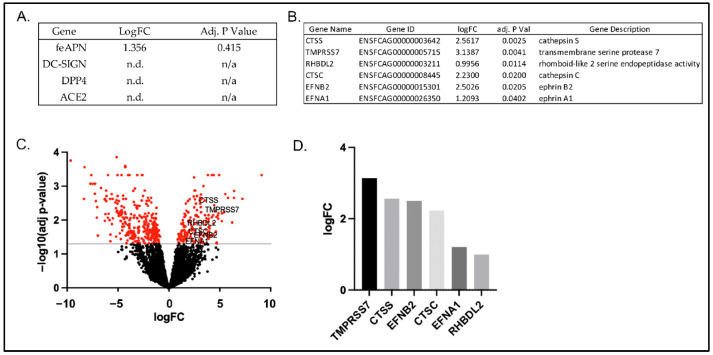
Genes of particular interest associated with coronavirus cell entry and/or pathogenesis. (**A**) Expression of known coronavirus cellular receptors detected or not detected in FCWF-4 CU and FCWF-4 ATCC cell lines. LogFC represents the log fold change of FCWF-4 CU gene expression relative to FCWF-4 ATCC gene expression. feAPN expression was detected in both FCWF-4 cell lines. (**B**) Six genes of interest to potentially explain differential FIPV serotype I replication. (**C**) Volcano plot of whole-transcriptome differentially expressed genes. The transcripts highlighted in red are significantly up- or down-regulated genes in FCWF-4 CU cells relative to FCWF-4 ATCC cells. Genes in (**B**) are specifically identified. (**D**) Bar graph depicting log fold change (logFC) of select genes in FCWF-4 CU cells relative to FCWF-4 ATCC cells.

In alignment with the targeted RT-qPCR findings, FCWF-4 CU and FCWF-4 ATCC cell lines both expressed feAPN mRNA, with FCWF-4 CU cells exhibiting a slightly higher transcription level for this receptor transcript, although not significantly greater than FCWF-4 ATCC cells (Figure 7A). Expression of the other three investigated coronavirus receptor transcripts (ACE2, DPP4, and DC-SIGN) was not detected in either cell line, in agreement with the result of the targeted RT-qPCR gene expression (Figure 4).

A detailed evaluation of the 458 significantly DE genes identified six genes of interest that might plausibly be associated with coronavirus cell entry and/or replication (Figure 7B). The expression of these six genes was significantly higher in FCWF-4 CU cells relative to FCWF-4 ATCC cells (Figure 7B–D).

Two of these genes of interest identified in FCWF-4 CU cells are *CTSS* and *CTSC*, cathepsins S and C, respectively. Cathepsins are cysteine proteases involved in the endosomal route of coronaviral cell entry via spike cleavage [58,59,60,61]. Two serine proteases were also identified. The first, *TMPRSS7*, or transmembrane serine protease 7, is part of a group of TMPRSS proteins associated with early coronavirus cell entry [62]. The other serine protease is *RHBDL2* (the rhomboid-like 2 serine endopeptidase). Although this serine protease is not known to be associated with coronavirus cell entry, some coronaviruses have been shown to utilize serine proteases for cell entry, making this serine protease worthy of further investigation [60,63,64].

The final two significantly upregulated genes possibly associated with coronavirus cell entry or pathogenesis are *EFNB2* and *EFNA1,* ephrin B2 and ephrin A1, respectively. These transcripts are of interest based on the fact that their encoded proteins can serve as entry receptors for a variety of viruses, including Hendra, Nipah, hepatitis C, and Epstein–Barr viruses [65,66,67,68]. These gene products have also been shown to serve as an alternative co-receptor for viral entry by SARS-CoV-2, SARS-CoV-1, and Middle Eastern respiratory syndrome (MERS)-CoV [69,70].

## 4. Discussion

In this study, we investigated the relative replication efficiencies of culture-adapted FIPVs I and II (Black I and WSU-79-1146) and the expression patterns of a selection of known coronaviral receptors in variably permissive feline cells. We found that feAPN, the FIPV II cellular receptor, is abundantly expressed in all four of the evaluated cell types, with CRFK cells demonstrating the highest level of expression. Although all of the evaluated cell types expressed feAPN, expression in feMDMs and both FCWF-4 cell types was approximately one to two logs less than in CRFK cells. We also found that all four of the examined cell types are permissive to FIPV II replication, consistent with the concept that feAPN serves as the FIPV II receptor. Prior studies have shown that although the receptor DC-SIGN is not required, it can serve as an optional co-receptor by enhancing FIPV II cell entry [34,70]. We only identified DC-SIGN expression in feMDMs, a cell type closely related to the natural host cell of FIPV in vivo. Our RNAseq data corroborates these gene expression findings in the two FCWF cell lines, with no transcripts detected for ACE2, DC-SIGN, and DPP4 and a slightly higher expression level of feAPN in FCWF-4 CU cells relative to FCWF-4 ATCC cells (although not significantly different).

DPP4, a type II transmembrane ectopeptidase, serves as the receptor for the zoonotic Middle Eastern respiratory syndrome coronavirus (MERS-CoV) [71]. Here, we detected DPP4 expression only in CRFK cells and, therefore, found no evidence to support DPP4 serving as a primary cellular receptor for FIPV I. ACE2, the cellular receptor for multiple coronaviruses, including HCoV-NL63, SARS-CoV-1, and SARS-CoV-2, is well documented in tissue and cellular distribution in humans and has been shown to be expressed in specific subpopulations of human macrophages [72,73,74,75,76]. Since FIPV infects macrophages in vivo and shares some pathogenic features similar to SARS-CoV-2, we reasoned that ACE2 expression should be examined in permissive feline cells and tissues. To our knowledge, there is an absence of peer-reviewed literature focused on ACE2 as a potential receptor for FIPV I. Using IHC, ACE2 expression was detected in a broad range of feline tissues and cell types, with the strongest staining intensity identified within a number of epithelial cells, including respiratory and intestinal epithelium. Interestingly, we detected ACE2 expression within the bronchiolar epithelium, while another recent study reported absent antigen detection for ACE2 within domestic cat bronchiolar epithelium [77]. Here, we further document a more extensive detailed list at both the tissue and cellular levels for ACE2 expression in cats, while previous studies have focused more on the evaluation of intestinal, kidney, and respiratory tissues [40,77,78]. ACE2 expression was also detected in scattered, individualized round cells in lymphoid tissues (lymph node and spleen specifically) within germinal centers and the paracortex and to a much less degree relative to DC-SIGN.

We found that Black I replicated most efficiently in FCWF-4 CU and CRFK cells; however, we did not identify ACE2 gene expression via RT-qPCR in any of the evaluated cell lines (or in the RNAseq data). Although, taken together, these results suggest that ACE2 does not serve as the primary cellular receptor for FIPV I, ACE2′s function as a co-receptor cannot be ruled out. It is worth noting that although we found CRFK cells to be permissive to Black I replication, there are experimental inconsistencies with regard to FIPV I replication in CRFK cells. Black (1980) initially reported CRFK permissibility to FIPV I (Black I), while Hohdatsu et al. (1998) were unable to infect CRFK cells with multiple strains of FIPV I (KU-2, Black I, UCD-1). Another study by Van Hamme et al. (2007) documented the inefficient attachment and internalization of Black I in CRFK cells [79]. A definitive explanation for these experimental differences is not available; however, it is possible these variable results relate to genetic changes in either the CRFK cell line or the Black I virus through prolonged subpassaging [19,21,79].

Expression of DC-SIGN, the co-receptor for FIPV II, was also examined using immunohistochemistry in feline tissues. It has been previously shown that antibody-mediated blockade of DC-SIGN greatly reduces FIPV I binding to feline blood monocytes [54]. In our in vitro experiments, we detected DC-SIGN expression only in fMDMs. Although these results suggest that DC-SIGN does not function as the primary receptor of FIPV I, a co-receptor function cannot be excluded. IHC-determined DC-SIGN expression in feline tissues was more limited than ACE2 expression and was predominantly associated with scattered individual round cells in lymphoid tissues, presumed macrophages, and dendritic cells, as discussed within the results. This is consistent with the expectation of histiocytic (dendritic cell and macrophage) expression of DC-SIGN [80].

The markedly different replication kinetics of Black I in the FCWF-4 ATCC and FCWF-4 CU cell lines (a 4-log difference) is interesting, considering that these cell lines are closely related (FCWF-4 CU is passage-derived from FCWF-4 ATCC). We reasoned that the (presumed) genetic similarities of these two feline cell lines and markedly differential viral growth kinetics might facilitate critical insights into FIPV serotype I replication. This was the impetus for pursuing global transcriptome analyses (RNAseq) of the two related cell lines. RNAseq data highlighted the overall similarities in gene expression patterns between the two cell lines, as expected, given their relatedness. RNAseq also identified 458 significantly DE genes. From this subgroup of genes, we identified six genes of particular interest to coronavirus biology.

Four of the six DE genes encode cellular proteases: two are cysteine proteases, cathepsins S (*CTSS*) and C (*CTSC*), and two are transmembrane serine proteases, transmembrane serine protease 7 (*TMPRSS7*) and rhomboid-like 2 serine endopeptidase (*RHBDL2*). Specific viral and host proteases are known to be essential for coronavirus replication and are involved in different steps of the virus life cycle, including cell entry, replication of the virus, protein maturation, and virion assembly [81]. TMPRSS7 is a member of a larger family of transmembrane serine proteases, one of which (e.g., TMPRSS2) is the dominant protease responsible for the proteolytic cleavage of SARS-CoV-2 at the cell surface, thereby triggering fusion of the viral lipid membrane with the host cell membrane [62]. Other transmembrane proteases associated with enhanced entry of SARS-CoV-2 include TMPRSS11D and TMPRSS13 [82]. Although TMPRSS7 has not been previously identified as a protease associated with coronavirus entry, its identification here warrants further investigation. To the authors’ knowledge, RHBDL2 has not been previously linked with viral cell entry or viral activation, but the DE of this protease in FCWF-4 CU cells warrants further investigation.

The two identified cysteine proteases are lysosomal peptidases (cathepsins), which are the main class of lysosomal peptidases [83]. Cathepsins are involved in virion entry and viral processing after endosomal uptake for a range of viruses, including reoviruses, Ebola virus, and coronaviruses, with cathepsin L being most commonly associated with the activation of coronaviral glycoproteins [84,85,86]. Of the two cathepsins identified in this research, cathepsin S has been associated with cleavage of the capsid protein during cellular entry in some strains of reovirus [87]. To the authors’ knowledge, cathepsin C has not been linked with viral entry or replication for any specific viruses to date; however, the widespread roles of cathepsins with other viruses make the upregulation of this gene in FCWF-4 CU cells notable and worthy of further investigation.

The remaining two DE genes of interest are ephrin B2 and ephrin A1. Ephrins (erythropoietin-producing hepatocellular carcinoma) are the largest family of receptor tyrosine kinases known amongst mammals [88]. Members of the ephrin and ephrin receptor binding pathways have been identified as receptors for multiple RNA and DNA viruses, including paramyxoviruses, flaviviruses, herpesviruses, and retroviruses [67]. Examples include Hendra and Nipah viruses (*Paramyxoviridae*), which utilize ephrin B2 and B3 as viral entry receptors [65,89], as well as the hepatitis C virus, which utilizes ephrin receptor A2 as a co-factor for cell entry [90]. Although ephrin usage by coronaviruses has not been previously reported in the peer-reviewed literature, a recent abstract reported an upregulation of ephrin A1 and B2 in the saliva of COVID-19 patients. The significance of this finding is presently unknown but is being explored as a biomarker for disease severity in COVID-19 patients [91].

Despite decades of study, multiple gaps in our collective understanding of FCoV biology and pathogenesis remain, particularly with regard to FIPV I, the predominant coronavirus in naturally occurring cases of FIP. A definitive determination of the FIPV I receptor, along with the phenotype of the viral host cell in cats, would likely facilitate the development of targeted treatments and shed mechanistic light on the varied clinical presentations of FIP. Recent success in propagating FIPV I (Black I) in FCWF-4 CU cells provides a tool to explore these questions. The six candidate genes identified in this research should be systematically evaluated for their biological relevance through targeted gene disruption, genetic transduction of non-permissive cell lines, antibody blockade, and pharmacologic inhibition of protease function. The data presented here provide a platform for further exploration of a small group of candidate receptors and proteases.

## Figures and Tables

**Table 1:** List of known alpha- and betacoronavirus cellular receptors.

Genus	Virus	Species	Receptor
*Alpha*	HCoV-NL63	Human	ACE2
	229E	Human	APN
	TGEV	Pig	APN
	PRCoV	Pig	APN
	FCoV Serotype I	Cat	ND
	FCoV Serotype II	Cat	APN, DC-SIGN
	CCoV	Dog	APN
*Beta*	SARS-CoV-1	Human	ACE2
	SARS-CoV-2	Human	ACE2, DC-SIGN
	MERS-CoV	Human	DPP4

ND = not determined; HCoV-NL63 = human coronavirus NL63; 229E = human coronavirus-229E; TGEV = transmissible gastroenteritis virus; PRCoV = porcine respiratory coronavirus; FCoV = feline coronavirus; CCoV = canine coronavirus; SARS-CoV-1 = severe acute respiratory syndrome-coronavirus-1; SARS-CoV-2 = severe acute respiratory syndrome-coronavirus-2; ACE2 = angiotensin-converting enzyme 2; APN = aminopeptidase N; DC-SIGN = dendritic cell-specific intercellular adhesion molecule-3 grabbing nonintegrin; DPP4 = dipeptidyl-peptidase 4 (DPP4).

**Table 2:** Primers utilized in all PCR reactions with annealing temperatures and expected amplicon sizes for each primer set.

Target		Sequence (5′-3′)	Annealing Temperature (°C)	Size (bp)
FIPV	Forward	GGAAGTTTAGATTTGATTTGGCAATGCTAG	58	112
	Reverse	AACAATCACTAGATCCAGACGTTAGCT		
GAPDH	Forward	AAATTCCACGGCACAGTCAAG	58	61
	Reverse	TGATGGGCTTTCCATTGATGA		
CD16	Forward	AACCAGCTTTCTGCTTCAGTATC	51	98
	Reverse	AGACCTGAGCGCATAGAGTATC		
ACE2	Forward	GACAACAGCCTGGAGTTTCT	51	134
	Reverse	GAGACGATGAGCAGGACAATAC		
DC-SIGN	Forward	GGCCTTTAAGATCCTGAGAGAGA	51	102
	Reverse	GCTGAGACCATCAACAGAAGAAG		
feAPN	Forward	GGTGTTTGACTCCATCTCCTAC	60	134
	Reverse	GGTAGATGGTGTTCCCGTATTT		
DPP4	Forward	ATGCAAGCACCGACTTCTAA	55	117
	Reverse	CGTAGCACCTCTAGCCATAAC		
FCoV I S For (lffs type I)	Forward	GTTTCAACCTAGAAAGCCTCAGAT	50	376
FCoV II S For (lcfs type II)	Forward	GCCTAGTATTATACCTGACTA		283
FCoV I and II S Rev (lubs)	Reverse	CCACACATACCAAGGCC		
nFCoV I S For (nlffles type I)	Forward	CCTAGAAAGCCTCAGATGAGTG	47	360
nFCoV II S For (nlcfs type II)	Forward	CAGACCAAACTGGACTGTAC		218
nFCoV I and II S Rev (nlubs)	Reverse	CCAAGGCCATTTTACATA		

**Table 3:** List of tissue- and cell-specific immunoreactivity for ACE2 and DC-SIGN in normal feline tissues.

Tissue	Cell	ACE2	DC-SIGN
Cerebrum	Neurons	0	0
	Glial cells	+0-2 (C)	0
Cerebellum	Purkinje cells	0	0
	Granular cells	0	0
Choroid Plexus	Ependymal cells	+1 (C)	0
Meninges	Fibroblasts	+1 (C)	0
Eye	Ganglion cells	+2 (C)	0
	Nuclear layer of retina	+0-1 (C)	0
	Fibroblasts	+2 (C)	0
	Pigmented epithelium	U	U
	Melanocytes	U	U
	Corneal epithelium	+0-1 (C)	0
	Keratocytes	0	0
	Ciliary body epithelium	+0-1 (C)	0
	Lens epithelium	+1 (C)	0
Optic nerve	Glial cells	+2 (C)	0
Thyroid	Follicular cells	+2-3 (C)	+2 (C)
	Parafollicular cells	+1 (C)	+0-1 (C)
Parathyroid	Chief and oxyphil cells	0	0
Adrenal gland	Cortex	+0-2 (C)	+1 (C; reticularis)
	Medulla	+1 (C)	0
Lymph node	Paracortex and follicles (scattered cells)	+2 (C)	+3 (C, M)
Adipose tissue	Adipocyte	+2 (C)	+1 (C)
Skeletal muscle	Myocyte	+1 (C)	0
Smooth muscle	Myocyte	+0-1 (C)	0
Tongue	Epithelium	+2-3 (C, M)	0
Esophagus	Epithelium	+2 (M)	0
Trachea	Airway epithelium	+2 (C)	0
	Submucosal gland epithelium	+2 (C)	0
Lung	Bronchial/bronchiolar epithelium	+2-3 (C)	+1-2 (C)
	Type I pneumocyte	+1-2 (U)	+1-2 (U)
	Type II pneumocyte	+2 (C)	+1 (C)
	Submucosal gland epithelium	+1-2 (C)	0
Heart	Myocyte	+0-1 (C)	+0-1 (C)
Vessels	Endothelium	+1-2 (C)	0
	Mural smooth muscle	+2 (C)	0
Liver	Hepatocyte	+1 (C)	0
	Biliary epithelium	0	0
Kidney	Cortical tubular epithelium	+3 (M, apical); +1 (C)	0
	Medullary tubular epithelium	+1 (C)	0
Spleen	Red pulp, scattered cells	+1-2 (C)	+2-3 (C)
	White pulp (rare)	0	+1 (C)
Pancreas	Acinar cells	+0-1 (C)	0
	Islet cells	0	0
Stomach	Mucosal epithelium	+2 (C)	0
Small Intestine	Mucosal epithelium, apical	+3 (M); +1 (C)	0
Ileum	Mucosal epithelium, apical	+3 (M); +1 (C)	0
Colon	Mucosal epithelium	+3 (C)	0
Intestine as a whole	Lamina propria, scattered cells	+3 (C)	+3 (C)

0 = absent to nonspecific, +1 = weak, +2 = moderate, +3 = strong, C = cytoplasmic, M = membranous, U = undetermined.

## Data Availability

The raw data supporting the conclusions of this article can be made available by the authors without undue reservation.
